# Physical inactivity and associated factors in Iranian children and adolescents: the Weight Disorders Survey of the CASPIAN-IV study

**DOI:** 10.15171/jcvtr.2017.06

**Published:** 2017-03-13

**Authors:** Roya Kelishadi, Mostafa Qorbani, Shirin Djalalinia, Ali Sheidaei, Fatemeh Rezaei, Tahereh Arefirad, Saeid Safiri, Hamid Asayesh, Mohammad Esmaeil Motlagh

**Affiliations:** ^1^Child Department of Pediatrics, Child Growth and Development Research Center, Research Institute for Primordial Prevention of Non-communicable Disease, Isfahan University of Medical Sciences, Isfahan, Iran; ^2^Non-communicable Diseases Research Center, Alborz University of Medical Sciences, Karaj, Iran; ^3^Chronic Diseases Research Center, Endocrinology and Metabolism Population Sciences Institute, Tehran University of Medical Sciences, Tehran, Iran; ^4^Non-communicable Diseases Research Center, Endocrinology and Metabolism Population Sciences Institute, Tehran University of Medical Sciences, Tehran, Iran; ^5^Development of Research & Technology Center, Deputy of Research and Technology, Ministry of Health and Medical Education, Tehran, Iran; ^6^Department of Epidemiology and Biostatistics, Shahid Beheshti University of Medical Science, Tehran, Iran; ^7^Department of Social Medicine, Medical School, Jahrom University of Medical Sciences, Jahrom, Iran; ^8^Department of Exercise Physiology, Science and Research Branch, Islamic Azad University, Tehran, Iran; ^9^Managerial Epidemiology Research Center, Department of Public Health, School of Nursing and Midwifery, Maragheh University of Medical Sciences, Maragheh, Iran; ^10^Department of Medical Emergencies, Qom University of Medical Sciences, Qom, Iran; ^11^Department of Pediatrics, Ahvaz Jundishapur University of Medical Sciences, Ahvaz, Iran

**Keywords:** Physical inactivity, Anthropometric Measures, Screen Time, Children and Adolescents

## Abstract

***Introduction:*** This study aims to assess the associated factors of physical inactivity among Iranian children and adolescents at national level. The second objective is to assess the relationship of physical inactivity with anthropometric measures.

***Methods:*** Along with a national surveillance program, this survey on weight disorders was conducted among a nationally-representative sample of Iranian children and adolescents, aged 6-18 years. Students were selected by multi-stage cluster sampling from rural and urban areas of 30 provinces of Iran. The Physical Activity Questionnaire for Adolescents (PAQ-A) was used to assess physical activity (PA). Using PAQ-A instrument, PA of past week categorized as; low PA level, that included those who scored between 1 to 1.9 on the PAQ-A instrument and high PA level that included participants with estimated scores between 2-5 PAQ-A.

***Results:*** Participants were 23183 school students (50.8% boys) with a mean age of 12.55 ± 3.3 years, without significant difference in terms of gender. Totally, 23.48% of participants (13.84% of boys and 33.42% of girls) were physically inactive. In multivariate logistic regression model, with increased age in children and adolescence, the odds of a physically inactivity increased (OR: 1.08; 95% CI: 1.07-1.10). The odds of prevalence of both obesity and underweight were high in children and adolescents with low PA. There was a decreasing trend in PA in higher school grades.

***Conclusion:*** We found a considerably high prevalence of physical inactivity in Iranian children and adolescents, with higher rates among girls and older ages. However, we did not find correlation between PA and socioeconomic status (SES). Because of the positive relationship between PA and ST, future studies should consider the complex interaction of these two items. Multidisciplinary policies should be considered in increasing PA programs among children and adolescents.

## Introduction

Worldwide, it is recognized that many children and adolescents have sedentary lifestyle.^1^ A growing body of evidence has documented many short and long-term adverse effects of children’s physical inactivity on their healthcare.^2-4^ Considering the fact that physical activity (PA) habits are established during childhood,^5^ determining the associated factors of physical inactivity and its consequences in children and adolescents can provide to boost public knowledge, especially among families and school-based policy makers about disadvantage of sedentary lifestyle in this age group.

On the other hand, PA is known as a complex and multi factorial behaviors, which is influenced by socio-demographic,^6^ psychological,^7^ physiological^8^ environmental and cultural variables^9^ Thus, identifying the effective factors of sedentary lifestyle in children and adolescents can help to design appropriate strategies for increasing PA among children and adolescents.

Evidence from most studies indicates that many Iranian children and adolescents have inadequate level of PA.^10,11^ For instance, based on pervious study the mean of vigorous PA in Iranian adolescents and children were 0.6 h/d.^12^ Furthermore, during last years, Iranian children and adolescents are facing high prevalence of obesity and risk factors of non-communicable diseases, with physical inactivity as one of their major etiologies.^12-14^

Previous studies focused on only a few associated factors with physical inactivity in Iranian children and adolescents^10,12,15,16^ whereas, the current study aims to determine the association of different factors, including socioeconomic status (SES), body image, and anthropometric indexes, with physical inactivity among children and adolescents at national level.

## Materials and Methods

Present paper discuss the findings of national survey of school students’ high risk behaviors of the school-based surveillance system entitled Childhood and Adolescence Surveillance and PreventIon of Adult non communicable disease (CASPIAN-IV) study^17^ and its complementary research on determinants of weight disorders.^18^

Benefitting from multi-stage cluster sampling methods, 23183 school students selected from elementary, intermediate and high schools of the rural and urban areas of 30 provinces of Iran. The age range was from 6 to 18 years. Other methods are illustrated in details throughout the study design.^1,2^

Demographic data and variables of interest were gathered through a validated questionnaires designed based on the World Health Organization Global School-based Student Health Survey (WHO-GSHS).^19^ In these processes at the first step; students and one of their parents were asked to response questions and at second step anthropometric measures were taken by train experts.

Family based characteristics including: family history of chronic diseases (hypertension, dyslipidemia, diabetes, and obesity), parental level of education (the highest total years of schooling), possessing a family private car and type of home (rented/owned), dietary behaviors, PA, and sedentary lifestyle were asked from all of participants or their parents.

Data and Safety Monitoring Board (DSMB) of the project was responsible to different levels of quality assurance and control that were followed through standardizes predefined protocols.^17^

Height (Ht) and weight (Wt) were measured, without shoes and with light clothing to the nearest 0.1 unit of correspond measure (cm for height and kg for weight). Body mass index (BMI) was calculated from weight and height [BMI = weight (kg) ⁄height (m^2^)].^20,21^ Waist circumference (WC) was detected over skin, midway between the lower border of the rib margin and the iliac crest at the end of normal expiration, to the nearest 0.1 cm. Both of WC and height were measured using a non-elastic tape. Abdominal obesity was defined as waist to height ratio (WHtR) more than 0.5.^2^ Neck circumference (NC) measured by the tape underneath the Adam’s apple at a comfortable position to the nearest 0.1 cm.^18^

Overweight and general obesity considered base on Centers of Disease Control and Prevention (CDC) and the percentiles computed in the population studied were used for the classification of the children and adolescents as overweight (85–94th percentile) and obese (> 95th percentile).^22^

### Estimation of socioeconomic status

Following analytical methods and variable selection of previous documented evidence in the Progress in the International Reading Literacy Study (PIRLS)^23^ Using principle component analysis (PCA), variables including parental education, parents’ job, possessing private car, school type (public/private), and having personal computer in home were summarized in one main component of SES. This main component was categorized into five classes, from them the first one was defined as a low SES, and at the descending trend fifth was recognized as a high level of SES.

### Screen time and physical activity

The average number of hours per day that students spent for each of components of; watching TV/VCDs, personal computer (PC), or electronic games (EG) during the entire week including weekends, were considered for estimation of screen time (ST) behavior in those specific fields. For the analysis of correlates of ST, according to the international ST recommendations, total cumulative spent time for ST was categorized into two groups of less than 2 h/d (Low) , and 2 h/d or more (High).^24,25^

The Physical Activity Questionnaire for Adolescents (PAQ-A) is a modified version of the Physical Activity Questionnaire for Children (PAQ-C) that developed for assessing the general levels of PA of children. In this approach a self-administrated questionnaire ask about 7-day recalls of sports or activities. These included sports or activities that led to heavy sweating or significant increase in respiratory and heart rate, or games that make participants breathe hard (such as skipping, running, and climbing ask). It also gather information on PA during spare time, physical education period and lunchtime, as well as after school, in the evenings and on weekends.^26,27^

Using PAQ-A instrument leisure time PA of past week (weekly frequency leisure time PA outside the school led to heavy sweating or large increases in breathing or heart rate) categorized as; low PA level, that included those who scored between 1 to 1.9 on the PAQ-A instrument and high PA level that included participants with estimated scores between 2-5 PAQ-A.^26,27^ The validity and reliability of questioner, in Iranian population, were approved through a comprehensive national study.^19^

### Statistical analyses

Quantitative variables are expressed as means ± standard deviation (SD) and qualitative variables as percentages. Differences between means were investigated by *t* test or analysis of variance (ANOVA) test and for categorized variables; the Pearson chi-square test used to comparison the percentages.

Logistic regression analyses were used to evaluate the association of mean of anthropometric measures according to PA categories. Adjusting was considered for age, living area, SES, FH of obesity, school type and ST.

Association of independent variables including residence area, sex, family history of obesity, age, body image, ST, SES quintile, abdominal obesity, and weight status, with physical inactivity were assess through logistic regression model.

Results of logistic regression are presented as odds ratio (OR) and 95% CI. In all analysis design of sampling (cluster sampling) were considered. All statistical analyses were performed using programs available in the STATA version 10. A *P* value of less than 0.05 was considered as statistically significant.

## Results

Participants of this national study were 23 183 school students (50.8% boys) with a mean age of 12.55 ± 3.3 years, without significant difference in terms of gender. Overall, 73.4% of participants were from urban areas and 26.6% from rural areas. Characteristics of the study participants are presented in Table 1 by gender and age group. Totally, 23.48% of participants (13.84% of boys and 33.42% of girls) were physically inactive. Among both girls and boys, younger age group (6-12 years) was more active than‏ the older age group (*P* < 0.001).

**Table 1:** Characteristics of study population according to sex and age: the CASPIAN IV

	**Girls**				**Boys**			
	**Total**	**6-12 years old**	**13-18 years old**	***P***	**Total**	**6-12 years old**	**13-18 years old**	***P***
n	11244	5336 (47.46%)	5908 (52.54%)	-	11597	5972 (51.50%)	5625 (48.50%)	-
Physical activity (%)								
‏ Inactive	33.42	26.87	39.34	<0.001	13.84	11.64	16.18	<0.001
‏ Active	66.58	73.13	60.66		86.16	88.36	83.82	
Screen time (%)								
TV								
< 2 h/d	30.13	29.15	31.03	0.035	29.68	29.92	29.42	0.563
≥ 2 h/d	69.87	70.85	68.97		70.32	70.08	70.58	
PC								
< 2 h/d	75.62	78.52	73.02	<0.001	83.94	84.09	83.79	0.705
≥ 2 h/d	24.38	21.48	26.98		16.06	15.91	16.21	
Total								
< 2 h/d	57.27	58.16	56.45	0.074	60.80	60.65	60.95	0.748
≥ 2 h/d	42.73	41.84	43.55		39.20	39.35	39.05	
Obesity (%)								
General								
No	94.72	94.88	94.57	0.453	92.26	91.28	93.30	<0.001
Yes	5.28	5.12	5.43		7.74	8.72	6.70	
Abdominal								
No	83.77	85.27	82.42	<0.001	81.09	81.39	80.77	0.395
Yes	16.23	14.73	17.58		18.91	18.61	19.23	
Overweight								
No	87.13	88.27	86.10	0.001	86.87	87.69	85.99	0.007
Yes	12.87	11.73	13.90		13.13	12.31	14.01	
School type								
Public	92.56	94.21	91.08	<0.001	89.25	90.35	88.09	<0.001
Private	7.44	5.79	8.92		10.75	9.65	11.91	
SES‏ quintile								
1	20.08	18.15	21.84	<0.001	20.45	17.57	23.49	<0.001
2	19.46	19.50	19.42		20.03	20.25	19.79	
3	20.42	20.36	20.47		19.78	20.23	19.31	
4	20.13	21.80	18.62		19.72	20.11	19.31	
5	19.90	20.19	19.65		20.03	21.84	18.11	

Overall, 69.87% and 70.32% of girls and boys watched TV more than 2 h/d, respectively. The majority of girls and boys (75.62% and 83.94% respectively) used PC for less than 2 h/d. In total, in both genders, no significant association was found between the age group and the time spent on ST. Overall, higher prevalence of obesity was observed in boys as compared to girls. Obesity was more prevalent in the older age group than the younger age group.

Comparison of anthropometric variables between the physically inactive and active groups is presented in Table 2. In crude model, in both genders, almost all anthropometric variables showed significant difference between the physically inactive and active groups. In general, the inactive group had higher weight, height and BMI, as well as waist, hip and NCs than the active group in both sexes. After adjustment for co-variables, only in girls BMI was higher in inactive group compare to active group (19.00 vs 18.12 kg/m^2^, respectively, *P* = 0.04). In both genders, inactive individuals had significantly higher BMI compared to their active counterparts (18.90 vs 18.67 kg/m^2^, respectively, *P* < 0.001). In total, the active group were taller than the inactive group (147.23 vs 146.81 cm, respectively, *P* = 0.03).

**Table 2:** Crude and adjusted mean of anthropometric measures according to physical activity categories: the CASPIAN IV study

	**Crude model**			Adjusted Model*		
	**Inactive**	**Active**	***P***	**Inactive**	**Active**	***P***
**Total**						
n (%)	5363 (23.48)	17478 (76.52)		5363 (23.48)	17478 (76.52)	
Weight (kg)	44.92 (0.23)	41.79 (0.13)	<0.001	42.26 (0.18)	42.14 (0.10)	0.57
Height (cm)	149.77 (0.24)	146.67 (0.14)	<0.001	146.81 (0.17)	147.23 (0.09)	0.03
BMI (kg/m^2^)	19.37 (0.06)	18.65 (0.03)	<0.001	18.90 (0.06)	18.67 (0.03)	<0.001
Waist (cm)	67.66 (0.16)	66.49 (0.09)	<0.001	66.27 (0.15)	66.68 (0.08)	0.02
WHtR	0.45 (0.001)	0.45 (0.0004)	0.16	0.45 (0.0001)	0.45 (0.0005)	0.20
Hip (cm)	83.21 (0.19)	80.33 (0.10)	<0.001	81.28 (0.16)	80.68 (0.09)	0.001
WHR	0.82 (0.002)	0.83 (0.001)	<0.001	0.82 (0.002)	0.83 (0.001)	<0.001
Wrist (cm)	15.29 (0.09)	15.44 (0.16)	0.61	15.06 (0.23)	15.37 (0.13)	0.24
Neck (cm)	30.71 (0.07)	30.27 (0.04)	<0.001	30.22 (0.08)	30.38 (0.04)	<0.09
**Boys**						
n (%)	1605 (13.84)	9992 (86.16)		1605 (13.84)	9992 (86.16)	
Weight (kg)	46.36 (0.48)	42.61 (0.18)	<0.001	43.88 ± 0.35	42.56 ± 0.14	<0.001
Height (cm)	152.34 (0.51)	147.93 (0.19)	<0.001	149.69 ± 0.33	148.10 ± 0.13	<0.001
BMI (kg/m^2^)	19.48 (0.08)	18.69 (0.05)	<0.001	18.71 ± 0.11	18.55 ± 0.04	0.16
Waist (cm)	68.81 (0.33)	67.41 (0.12)	<0.001	67.57 ± 0.29	67.36 ± 0.11	0.49
WHtR	0.45 (0.002)	0.46 (0.001)	0.04	0.45 ± 0.002	0.46 ± 0.001	0.12
Hip (cm)	81.56 (0.35)	79.83 (0.13)	<0.001	80.15 ± 0.29	79.88 ± 0.12	0.37
WHR	0.85 (0.003)	0.85 (0.001)	0.84	0.85 ± 0.004	0.85 ± 0.002	0.95
Wrist (cm)	15.86 (0.18)	15.41 (0.06)	0.01	15.55 ± 0.19	15.44 ± 0.07	0.57
Neck (cm)	31.50 (0.15)	30.66 (0.06)	<0.001	31.06 ± 0.16	30.71 ± 0.06	0.04
**Girls**						
n (%)	3758 (33.42)	7486 (66.58)		3758 (33.42)	7486 (66.58)	
Weight (kg)	44.30 (0.25)	40.69 (0.18)	<0.001	41.92 ± 0.19	41.39 ± 0.14	0.03
Height (cm)	148.68 (0.25)	144.99 (0.19)	<0.001	146.08 ± 0.18	145.81 ± 0.13	0.24
BMI (kg/m^2^)	19.48 (0.08)	18.69 (0.05)	<0.001	19.00 ± 0.07	18.12 ± 0.05	0.04
WC (cm)	67.18 (0.18)	65.26 (0.13)	<0.001	65.91 ± 0.17	65.67 ± 0.12	0.26
WHtR	0.45 (0.001)	0.45 (0.001)	0.12	0.45 ± 0.001	0.45 ± 0.001	0.57
Hip circumference (cm)	83.91 (0.22)	81.02 (0.16)	<0.001	81.90 ± 0.19	81.69 ± 0.13	0.37
WHR	0.81 (0.002)	0.81 (0.001)	0.32	0.81 ± 0.002	0.81 ± 0.001	0.08
WC (cm)	15.04 (0.11)	15.48 (0.37)	0.41	14.89 ± 0.37	15.27 ± 0.26	0.42
NC (cm)	30.37 (0.08)	29.76 (0.07)	<0.001	29.95 ± 0.08	29.88 ± 0.06	0.56

Abbreviations: BMI, body mass index; WC, waist circumference; NC, Neck circumference; WHtR, waist to height ratio (WHtR); * Adjusted for age, living area, SES, FH of obesity, school type and screen time; WHR, waist to hip ratio.

As presented in Table 3, the logistic regression in the crude model showed that compared to rural residents, urban residents were less likely to be physically inactive (crude OR: 0.79; 95% CI: 0.74-0.85; *P* < 0.001) while after adjustment, this difference was no more statistically significant (OR: 0.95; 95% CI: 0.86-1.04, *P* = 0.95). Moreover, in comparison to girls, boys were less likely to be physically inactive (adjusted OR: 0.33; 95% CI: 0.31-0.36; *P* < 0.001). With increasing age among participants, the OR of PA decreased significantly (adjusted OR: 1.08; 95% CI: 1.07-1.10; *P* < 0.001).

**Table 3:** Association of independent variables with physical inactivity in logistic regression model: the CASPIAN IV study

	**Crude OR**	**95% CI**	**P-value**	**Adjusted OR**	**95% CI**	**P-value**
Region (rural)						
Urban	0.79	0.74-0.85	<0.001	0.95	0.86-1.04	0.27
Sex (female)						
Male	0.32	0.30-0.34	<0.001	0.33	0.31-0.36	<0.001
FH of obesity (No)						
Yes	1.05	0.98-1.13	0.17	1.06	0.96-1.16	0.24
Age (year)	1.09	1.08-1.10	<0.001	1.08	1.07-1.10	<0.001
Body image (normal)						
Tiny	1.05	0.98-1.12	0.17	1.11	1.02-1.22	0.01
Fat	1.00	0.92-1.09	0.94	1.03	0.92-1.15	0.60
TV (≤2 h)						
>2 h	0.88	0.82-0.94	<0.001	0.88	0.81-0.96	0.004
PC(≤2 h)						
>2 h	0.85	0.78-0.93	<0.001	0.72	0.65-0.79	<0.001
SES quintile (1)						
2	0.94	0.84-1.04	0.20	0.96	0.85-1.09	0.54
3	0.95	0.85-1.05	0.33	0.95	0.84-1.08	0.48
4	1.02	0.92-1.13	0.76	1.03	0.91-1.17	0.65
5	1.04	0.94-1.16	0.43	1.09	0.95-1.24	0.23
Abdominal obesity (No)						
Yes	1.02	0.94-1.10	0.65	0.92	0.81-1.05	0.23
Weight status (normal)						
Underweight	1.05	0.95-1.17	0.31	1.14	1.01-1.30	0.04
Overweight	1.06	0.97-1.16	0.19	1.11	0.97-1.26	0.13
Obesity	0.97	0.86-1.10	0.67	1.22	1.01-1.48	0.04

In comparison with participants who watched TV less than 2 h/d, those who watched TV or used personal computer (PC) more than 2 h/d were more likely to be physical active (adjusted OR: 0.88; 95% CI: 0.81-0.96; *P* = 0.004 and adjusted OR: 0.72; 95% CI: 0.65-0.79; *P* < 0.001 respectively ). The risk of obesity and underweight were high in children and adolescents with low PA (adjusted OR: 1.22; 95% CI: 1.01-1.48; *P* = 0.04, and adjusted OR: 1.14; 95% CI: 1.01-1.30; *P* = 0.04, respectively).

**Figure 1:**
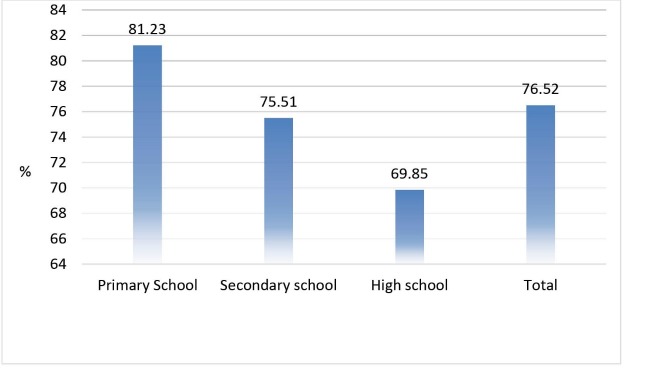
Prevalence of physical activity according to the school grade: the CASPIAN IV study

Figure 1 shows the status of PA at different school levels. A decreasing trend in PA was documented by increasing school grades. Accordingly, primary school students were more active (81.23%) than secondary school students (75.51%) and secondary school students were more active than high school students (69.85%).

## Discussion

To the best of our knowledge this study is the first studies that assess the physical inactivity and associated factors in a national representative sample of Iranian children and adolescence.

Consistent with several previous investigations, present study showed a considerable high prevalence of physical inactivity in Iranian children and adolescents (23.48%). PA level was about 20% higher in boys than in girls.^11,28^ The underlying factors for gender difference in PA looked from different points of views. It is suggested that boys are more encouraged and supported by family, friends and coaches to be active.^11,29,30^ Findings of another study, in terms of behavioral and psychological aspects, suggested that boys are willing participants in larger groups and engage in more active games than girls whereas, girls tend to take part in smaller groups and participate in passive and verbal games.^31^

Studies show that media have a great role in increasing unhealthy life style habits mostly happen during watching TV or movies.^11,29,30^

Our findings showed a decline in PA in the transition from childhood to adolescence. Although similar trends were observed in related studies in the pediatric age group, factors associated with this change should be more studied.^32-34^ A study suggests that the reasons for this decline are due to decreases in the number of attractive and available physical activities and the short time spent for each activity.^35^ Experimental studies emphasize that age-related decline in PA might have a biological basis, and the role of dopamine function in occurrence of this phenomenon must be more discussed^36^ Age-related decline in PA as well as increased time spent for school homework might be of main causes for the escalating trend of physical inactivity from primary school to high school.^37^ Moreover, in the current study, decline in PA with increased age was greater for girls than boys. This finding is consistent with some previous investigations.^38,39^ However, factors associated with this phenomenon are still not well understood. It is also suggested that decline in PA is associated with biological maturity, which occurs earlier in girls than in boys.^40^

Our findings are consistent with previous studies that showed higher BMI values in inactive individuals.^3,41,42^

In the current study, no significant association existed between SES and physical inactivity. This finding is consistent with a study conducted in children and adolescents aged 3-18.^43^ However, in contrast, another study provided evidence for a positive relationship between PA and SES in children and adolescents aged 4–18.^44^ A study on children and adolescents, aged 3-18 years, showed negative relationship between PA and SES.^45^ This variety in results can be likely because of cultural differences in various communities.

Our findings revealed that children and adolescents who spent prolonged time watching TV and using PC were more likely to have PA levels. A study showed similar results in which using computer was related to increased PA while, watching television was not related to decreased PA.^45^ On the other hands, some studies found decreasing PA with increasing duration of watching TV.^46,47,48^ It seems that the relationship between ST and PA is complex; and in studies on activity of children and adolescents, these two items should be considered separately.

Study limitations and strengths: The main limitation of present study it cross- sectional nature, which preclude causal interpretation of findings and some limitation of recall bias of participants in some information. The main strengths of this study are its novelty in the pediatric age group and covering a large and nationwide sample size and also using validated questionnaire for assessing PA.

## Conclusion

We found a very high prevalence of physical inactivity in Iranian children and adolescents, with higher rates among girls and older ages. However, we did not document any association between PA and SES. Because of the positive relationship between PA and ST, future studies should consider the complex interaction of these two items. Multi-dimensional policies should be considered in increasing PA programs among children and adolescents.

## Ethical approval

The project run with an interactive partnership between the Ministry of Health and Medical Education; Ministry of Education and Training, Child Growth and Development Research Center, Isfahan University of Medical Sciences, and Alborz University of Medical Sciences. Verbal and written consent were obtained from all of participants and one of their parents, ethical approval obtained from ethical committees of Isfahan and Alborz universities of Medical Sciences.

## Competing interests

The authors declare that they have no competing interests.

## Funding

This project is a collaborative effort between the School Health of Ministry of Health and Medical Education, Isfahan University of Medical Sciences and Alborz University of Medical Sciences.

## Acknowledgments

The authors are thankful of the large team working on this study and all participants in different provinces.
